# Poly(Lactic Acid)-Based Nanobiocomposites with Modulated Degradation Rates

**DOI:** 10.3390/ma11101943

**Published:** 2018-10-11

**Authors:** Iozzino Valentina, Askanian Haroutioun, Leroux Fabrice, Verney Vincent, Pantani Roberto

**Affiliations:** 1 Department of Industrial Engineering, University of Salerno Via Giovanni Paolo II, 132, 84084 Fisciano (SA), Italy; viozzino@unisa.it; 2 Institut de Chimie de Clermont Ferrand (ICCF), UMR 6296 Université Clermont Auvergne, CNRS, Sigma Clermont, ICCF, F-63000 Clermont-Ferrand, France; haroutioun.askanian@sigma-clermont.fr (A.H.); Fabrice.Leroux@uca.fr (L.F.)

**Keywords:** lifetime durability, degradation, poly(Lactic Acid), layered double hydroxides, nanobiocomposites

## Abstract

In the field of biodegradable polymers such as poly(Lactic Acid) (PLA), it is quite well known that their kinetics of hydrolysis strongly depend on the pH of the hydrolyzing medium. The idea explored during this study focused on PLA, is the addition of additives that are able to control the pH of water when it diffuses inside the polymer. For instance, acids (i.e. succinic acid, also used as food additive) are bio- and eco- friendly additives that are able to play this role. In order to control the release of these molecules and their dispersion inside the polymer, their intercalation in biocompatible nanofillers like layered double hydroxides (LDH) is here considered. The additives have been dispersed in the polymer by melt compounding, commonly used in the plastic industry. Several composites of PLA (4032D) and LDH intercalated with organic acids (succinic, fumaric, and ascorbic acid) have been obtained by an extrusion process. From all extruded materials, PLA films obtained by compression molding were then subjected to hydrolysis tests. The results showed that the mentioned molecules, dispersed in the polymer, are able to control the rate of hydrolysis, and experimental results show an increase of degradation time for samples containing LDH-organic acid (in particular with LDH-succinic acid), making such hybrid additives an appropriate and efficient solution for PLA.

## 1. Introduction

The growing attention to biodegradable polymers relies on several aspects: rising environmental concerns from consumers, who are increasingly willing to pay higher prices for green products [1], and compostability as an alternative end-of-life option, with legislative drivers and with specific functionality of certain bioplastics contribute to the increasing interest [2].

The transition from a fossil-based economy to a bio-based economy is an important concern.

Poly(Lactic Acid), (PLA) [3], an aliphatic thermoplastic polyester produced from renewable sources, is one of the most attractive biodegradable polymers. Recent developments of the continuous process of production of PLA have the potential effect of lowering its price to be competitive with other degradable polymers, and potentially competitive today with petroleum-derived plastics.

PLA is used in many areas such as biomedical, packaging and tissue engineering, and there are many companies that are interested in its properties, mainly regarding its biodegradability. Therefore, it is very important to better understand the degradation characteristics of PLA, for both consumers and biomedical applications (due to its biocompatibility and bioresorbability).

As with other bioplastic materials, PLA shows: a limited processing window due to thermomechanical degradation [4,5,6], and a loss of thermo-mechanical properties when heated or exposed to humidity [7,8].

The propensity to degradation with loss of thermomechanical resistance, as well as limited gas barrier properties of PLA, significantly limits its specific industrial applications, particularly for durable products with long-term performance such as in the automotive, electronics, biomedical, and agriculture [9,10,11]. Indeed, there are cases in which the acceleration of degradation is desirable, while in other cases it is required to extend the service life of PLA, depending on the field of application.

In the natural environment, the degradation of PLA proceeds either through hydrolytic or enzymatic chain scission of ester bonds to produce low molecular weight oligomers and monomers, thus enabling assimilation by microorganisms. The biodegradation of PLA proceeds readily in a compost environment. In particular, abiotic hydrolysis was suggested as a major depolymerization mechanism, as well as being the rate-controlling step of PLA biodegradation in compost [12]. Therefore, any factor affecting the rate of hydrolysis could either accelerate or delay the whole biodegradation process.

Many studies have reported on the hydrolytic degradation of PLA, analyzing the factors that can influence such phenomena [13,14]. It has been demonstrated that hydrolysis of PLA is auto-catalytically accelerated through their carboxyl end groups [15,16], and that the pH of the degrading medium determines the kinetics [17]. However, the mechanism of hydrolysis is still not clear, since it is reported that random chain scission takes also place in acid [18] or in basic [19] conditions. Chain scission process can induce a much faster reduction of the physical properties [20] rather than end scission. Also, the effect of the stereochemical composition is not fully understood [21].

The possibility of controlling the rate of biodegradation is strategic toward the application of biodegradable polymers in many sectors [21,22].

The rate can be modified using several techniques such as blending, copolymerization, and addition of specific fillers. Blending polymers are a way to modify the physical and mechanical properties of polymers and then their degradability. Arias et al. [23] have controlled and predetermined the degradation profiles of poly(l-Lactic Acid)-based materials during hydrolytic degradation thorough melt-blending with different polyesters. However, this route changes the physical desired properties of PLA.

The use of additives to tune the rate of hydrolysis should preserve the intrinsic properties of PLA. Recently, Stloukal et al. [11], investigated the stabilization effect of a commercially available aromatic carbodiimide-based anti-hydrolysis agent (which can modify the diffusion of water into the polymer matrix), which is intended to improve the hydrolysis resistance of PLA-based materials and prevent their degradation during processing. Even more recently, Benali et al. [4] used silanized zinc oxide nanofillers (ZnOs) to tune the hydrolytic degradation of PLA. Other fillers such as clays [22,24] silver [25], SiO_2_ [26], and graphene [27] were evaluated. Apart from the results reported by Stloukal et al., which show a quite significant delay in hydrolysis, the use of other additives appear to be marginal, if not negative toward the stabilization of PLA [28].

This new field of research is only just beginning, and it is extremely promising.

The aim of this work is to control the degradation rate of PLA for obtaining plastic products that degrade in a time that is compatible with the application for which they are used. The route adopted is the combination of a two-dimensional layered material and an intercalation technique, which offers a new area for developing inorganic/organic nanohybrids with desired functionalities. In particular, layered double hydroxides (LDHs) are also called anionic clays, were adopted. This class of fillers is extremely interesting, since negatively charged molecules can be incorporated between hydroxide layers as charge compensating anions through ion exchange techniques [29]. These molecules can impart particular properties to the polymer matrix. LDHs can be represented by the general chemical formula [M^II^_1−x_M^III^_x_(OH)_2_]^x+^ A^n−^_x/n_·mH_2_O, where: M^II^ and M^III^ are the divalent and the trivalent cations respectively, A^n−^ are the exchangeable intralayer anions whose function is to balance an excess positive charge induced by cations. In this work, M^II^ and M^III^ were represented by Mg^2+^ and Al^3+^ respectively, while A^n−^ were organic anions deriving from the organic acids mentioned in the previous section. A review of the state-of-the-art techniques concerning PLA–LDH nano(bio)composites can be found in recent literature [29,30]. Despite numerous studies on the PLA–LDH systems, only a few papers deal with the effect of the presence of LDH on the degradation of PLA. Zhou and Xhantos [31] found a reduction of the degradation rate in PLA containing 5% of calcinated LDH, which was ascribed to the reduction of the catalytic effect of the carboxylic end groups induced by the filler. Eili et al. [32] observed an increasing biodegradation rate of nanocomposites of PLA and stearate–Zn–Al–LDH on increasing the filler content, possibly due to an increased water sorption. Oyarzabal et al. [33] studied the hydrolytic degradation of a nanocomposite of PLA and LDH modified with 4-biphenyl acetic acid (BPh), and observed that the filler inhibited the degradation rate at the early stages, due to the reduction of the diffusion of the oligomers resulting from hydrolysis, but then promoted a faster weight loss afterwards.

Clearly, as expected, the degradation rates of PLA–LDH nanobiocomposites heavily depend strongly on the incorporated organic molecules. Obviously, in order to be of interest for PLA, this molecule should be environmentally friendly, possibly bio-compatible, and it should not induce degradation in the molten state.

In this work, the hydrolytic degradation of nanobiocomposites of PLA and LDH of cation composition Mg_2_Al organo-modified with fumaric acid, succinic acid, and ascorbic acid was studied.

## 2. Materials and Methods

### 2.1. Materials

In this work, a PLA grade produced by Natureworks (Minnetonka, MN, USA) was used: 4032D (with about 2% of d-lactide).

The fillers used were: organic acids all received from Sigma-Aldrich (St. Louis, MO, USA) (succinic acid (1,4-Butandioic Acid), HOOC-CH_2_-COOH, E363), fumaric (trans-butenedioic, HOOC–CH=CH–COOH, E297) and ascorbic acids (oxo-3-gulofuranolactone, C_6_H_8_O_6_, E300)) and LDHs of cation composition Mg:Al (2:1) intercalated with the three organic acids just mentioned, used as host molecules. The selected organic acids were from natural sources and were biocompatible. They have the common characteristics to remain in solid state at the processing temperatures of PLA. Fumaric, succinic, and ascorbic acids were used as received from the supplier.

LDHs intercalated with organic acid were synthesized through a process in a dedicated section below. Magnesium nitrate, aluminum nitrate, and sodium hydroxide, used for the synthesis, were purchased from Sigma Aldrich (St. Louis, MO, USA).

### 2.2. Methods

#### 2.2.1. Synthesis of Layered Double Hydroxide

The LDHs were synthesized by co-precipitation. A metallic solution was prepared using the nitrate of the two cations considered, Mg(NO_3_)_2_ and Al(NO_3_)_3_. The quantities of the salt used were carefully calculated in order to maintain the Mg/Al ratio equal to 2 (a common ratio straightforward to prepare and present in the hydrotalcite mineral); in particular, 28.36 g of Mg(NO_3_)_2_ and 20.72 g of Al(NO_3_)_3_ were used. The salts were placed into a small vessel and 100 mL of distilled water was added, then the solution was stirred for 2 min to ensure the complete solubilization of the salts. an alkaline solution was also prepared by dissolving 8 g of sodium hydroxide (NaOH) in 100 mL of distilled water.

The synthesis reaction of LDHs was carried out in a 1 L volume reactor. The process was performed under a nitrogen atmosphere, in order to avoid any contamination by carbonation, at 25 °C and at atmospheric pressure. During the synthesis reaction of the LDHs, pH was continuously controlled and kept constant and equal to 9.5, which was found to be an optimal value to yield the present organic inorganic materials.

Organic acid, about 13 g, was directly inserted in the reactor and dissolved in 500 mL of distilled water. The system was covered and continuously stirred. The metallic solution and the alkaline solution were slowly pumped into the reactor. The flow rate of the metallic solutions was 0.5 mL/min; a pH meter and a pump were connected to a computer where a software program analyzed the pH value and transmitted to the pump the correct flow rate of the alkaline solution in order to keep the pH value constant.

Once the metal salts were depleted, the final solution in the reactor was stirred for three hours. After aging, the material was washed with distilled water and centrifuged (4000 rpm) three times, to be sure that all impurities were removed from the products of the reaction. The presence of highly crystallized salt is easily observed by XRD (Figure S1 in Supplementary Materials). Finally, the products were dried in oven for 24 h at 60 °C. After drying, whitish powders were obtained after grinding.

#### 2.2.2. Samples Production

The pellets of PLA, organic acids, and the LDHs synthesized were dried under a vacuum at 60 °C in an oven for 24 h. Dried materials were melt compounded in a Minilab Haake Thermo Scientific twin-screw mini-extruder (Waltham, MA, USA), with counter-rotating screws, at a homogeneous temperature of 170 °C, at 100 rpm, with a cycle time of 5 min and an average charge time of 4–5 min. The extrusions were carried out using dry nitrogen as the purge gas in the hopper. Several blends were studied, all reported in Table 1.

Several amorphous films were obtained from extruded materials by compression molding using a carver laboratory press at 170 °C with a time of 20 min. From these films of thickness of about 250 µm, samples were cut with dimensions of 1 cm × 1 cm, to be subjected to hydrolysis tests.

#### 2.2.3. Hydrolysis Tests

As mentioned above, PLA hydrolysis is the main mechanism of depolymerization, and the controlling step of the biodegradation process in compost [21]. Therefore, the rate of hydrolysis gives a direct indication of the rate of degradation during composting. Hydrolysis was conducted in distilled water, with a pH value of about 6.5. The tests were performed at 58 °C, which is the temperature adopted for biodegradation tests according to ASTM and ISO standards. Several samples of all the composites analyzed were placed in a glass container, filled with distillated water; the ratio between the amount of water (mL) and the mass of the dried sample (g) was set at 800. In order to keep a constant temperature of 58 °C during the hydrolysis test, a thermostatic bath with lid was used. Every 24 h the liquid was replaced by fresh distillated water with the same amount as the previous one: the glass vessel was emptied using a syringe equipped with a needle with 0.2 mm diameter. At pre-established times, all the samples in their vessels were dried under vacuum at 60 °C for about 3 h and weighted. After these operations, one of the samples was kept dried for further analysis. At the end, several vessels were placed again into the thermostatic bath. The samples during the hydrolysis test never left their vessels, in order to avoid undesirable hydrolyzed material losses. The hydrolysis process of all samples was followed for a maximum time of 60 days: after this, some samples were reduced into very small fragments and their mass became very small, for this reason it was very difficult to carry out analyses correctly. It is worth mentioning that according to the literature [21], the rate of hydrolysis at 58 °C is about 3–4 orders of magnitude larger with respect to the rate measured at room temperature. This means that 60 days at 58 °C would provide indications concerning the behavior of the samples for several years at lower temperatures. 

#### 2.2.4. Gel Permeation Chromatography (GPC Analysis)

GPC analyses were performed in order to evaluate the variations of molecular weight of the samples as hydrolysis proceeded. The equipment used to carry out the GPC measurements was a high performance liquid chromatography (HPLC) Waters system (Milford, MA, USA), equipped with an auto-sampler. Analyzed samples at different hydrolysis times were dissolved in tetrahydrofuran (THF), with a ratio (sample mass/solvent) equal to about 1/1 (g/mol) at 50 °C. The obtained solutions were then filtrated by using a filter Chromafil PTFE 0.45 µm.

#### 2.2.5. Differential Scanning Calorimetry (DSC Analysis)

DSC analyses were performed in order to assess the crystalline degree and the glass transition temperature of the different samples at selected times of hydrolysis. The tests were carried out by means of a DTA Mettler Toledo (DSC 822) (Columbus, OH, USA) under a nitrogen atmosphere. The samples, with a mass of about 5 mg, were subjected to the following thermal protocol:

Heating step from −10 °C to 200 °C at 10 °C/min (first heating);

Isothermal step for 5 min at 200 °C;

Cooling step from 200 °C to −10 °C at 10 °C/min (cooling);

Heating step from −10 °C to 200 °C at 10 °C/min (second heating).

The crystallinity degree was calculated from the thermograms obtained during the first heating scan according to the formula:

(1) $$Xc\left(t\right)=\frac{\int_{0}^{t}\frac{\delta Q}{\delta t}dt}{\int_{0}^{t\infty }\frac{\delta Q}{\delta t}dt}=\frac{\Delta H\left(t\right)}{\Delta H\infty }$$

in which *δQ*/*δt* is the heat flow measured by the calorimeter, Δ*H* is the integral of the heat flow after the baseline subtraction, and Δ*H*_∞_ is the latent heat of crystallization of a fully crystalline PLA, which was found in the literature to be 93 J/g [27]

#### 2.2.6. Rheological Tests (Hydrolysis at High Temperature Analysis)

In order to assess the significance of hydrolysis in the molten state, time sweep tests at constant frequency were performed [34,35]. The rheological tests were carried out by a Haake Mars II (Thermo scientific, Waltham, MA, USA) rotational rheometer in dynamic mode in a parallel plate configuration under nitrogen atmosphere. The diameter of the plates used was 20 mm with a gap of 0.45 mm. Rheological tests were performed at 200 °C, with a stress set to 500 Pa and a frequency of 1 rad/s, maintaining all values constant for 3 h. The materials, in order to evaluate the effect of the presence of water, were not dried before the tests. The rheological data will be reported in the plots versus the whole time at test temperature, including the time needed to set the gap.

#### 2.2.7. Mechanical Tests

Penetration mechanical tests were carried out in order to evaluate the breaking strength of the samples as hydrolysis proceeds. The tests were carried out using a DMA 8000 Perkin Elmer (Waltham, MA, USA).

**Figure 1:**
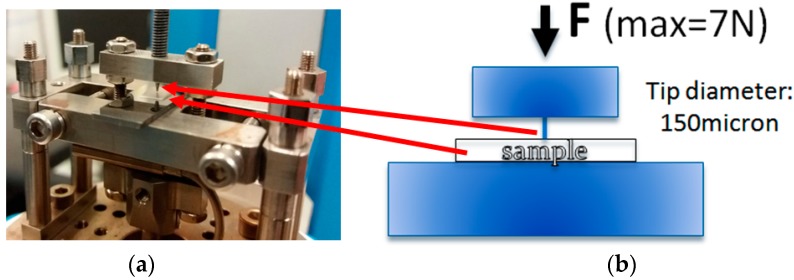
(**a**) Assembly for penetration test; (**b**) Scheme of the test.

A tip with a diameter of 150 μm was used and with a maximum force of 7 N, with a speed of 0.2 N/min. The operating scheme of the apparatus is shown in Figure 1.

The sample was placed under the tip, the latter moving until contacting the sample, and further provoking deformations and eventually breaks when the sample was more fragile than the maximum allowed value of 7 N (instrumental limit).

## 3. Results and Discussion

### 3.1. Benchmark LDH Selection

In this part of study, 4032D was used as the PLA matrix. Two different kind of pure LDHs were considered: one with NO_3_− as the interlayer anion, and the other one with CO_3_− as the interlayer anion.

PLA was added to the LDHs by melt compounding, as explained previously. The amount of LDH used was 3% (*w*/*w*). Three different samples were considered:

4032D

4032D+3%(LDH-CO_3_)

4032D+3%(LDH-NO_3_)

These samples were subjected to the hydrolysis process in the solid state at 58 °C; the experimental results are shown below.

#### 3.1.1. Hydrolysis at High Temperature (Rheological Analysis)

**Figure 2:**
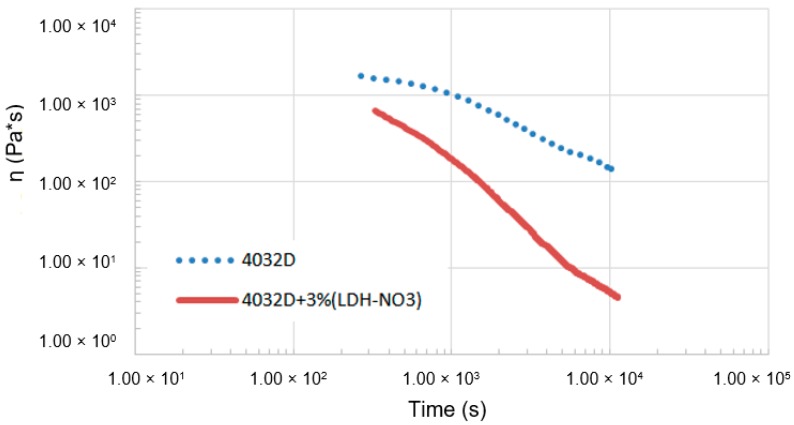
Time evolution of the complex viscosity during rheological tests of not hydrolyzed samples (4032D and 4032D + 3% LDH-NO_3_). The time on the horizontal axis includes the period needed to set the gap for each sample.

In order to assess the significance of hydrolysis in the molten state, time sweep tests were performed on 4032D and on 4032D + 3% LDH-NO_3_. The results of rheological analysis are reported in Figure 2.

From the data reported above, it can be observed that both materials presented a decrease of viscosity during time, due to thermal and hydrolytic degradation. However, the presence of LDH enhanced the degradation rate in the molten state significantly. This significantly limited the application of this filler in melt compounding.

#### 3.1.2. Weight Loss

**Figure 3:**
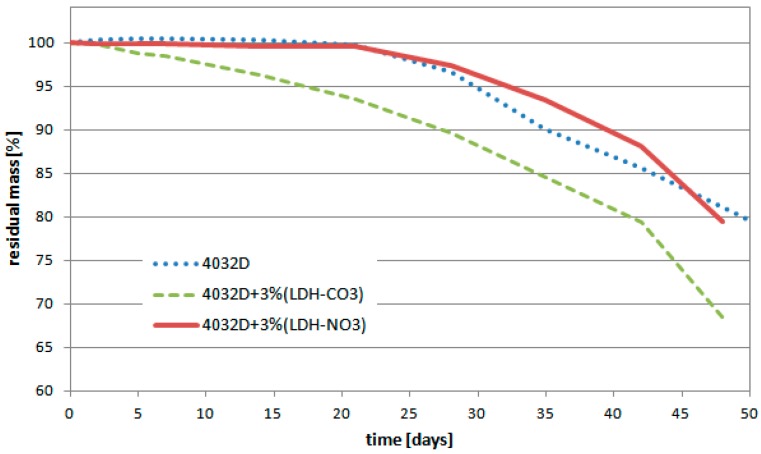
Weight loss during hydrolysis process (PLA and PLA + LDH-NO_3_ or -CO_3_).

In Figure 3, the variation of the sample mass as the hydrolysis process proceeded is reported. When H_2_O penetrates the polymer, hydrolytic degradation commences, converting the very long polymer chains into shorter water-soluble fragments. When these fragments leave the samples, a loss of weight of the samples is then detected.

Figure 3 shows that the presence in PLA of LDH-CO_3_ reduced the stability of the material with respect to hydrolysis. The composite with LDH-NO_3_, instead demonstrated a behavior that very similar to pure PLA.

#### 3.1.3. Calorimetric Analysis (DSC)

**Figure 4:**
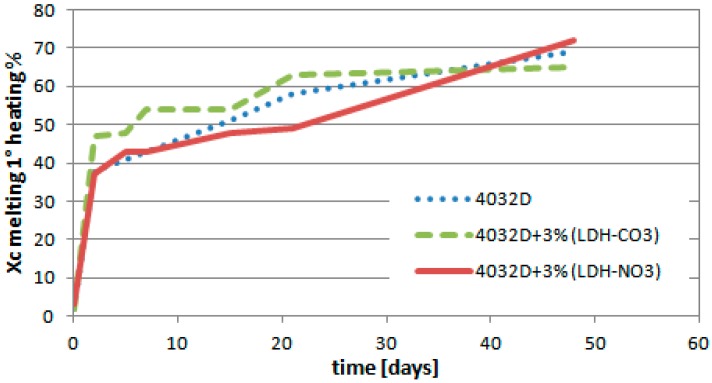
Variation of crystallinity degree during hydrolysis process (PLA and PLA + pure LDH/nitrate or carbonate).

In Figure 4, the evolution of the crystallinity degree as a function of hydrolysis over time is reported.

The degree of crystallinity (Xc) increased upon increasing the hydrolysis time. These crystallinity degrees were very high if compared with the maximum value that was reachable by the same materials as a consequence of thermal treatments (less than 45%). This was partly due to the erosion of the amorphous segments, but is was evidently due to the crystallization of the amorphous phase, since most of the samples were amorphous when starting the test.

As mentioned above, Xc increased for all the samples. There are, however, some differences between the samples. Indeed, up to 40 days, 4032D + 3% (LDH-CO_3_) exhibited that was Xc higher than the other two samples, probably indicative of a stronger degree of hydrolysis occurring in that case, and in agreement with a larger weight loss (Figure 3). In opposition, the lowest value of Xc was measured for 4032D + 3% (LDH-NO_3_), which presented relatively better resistance to the hydrolysis process. In Table 2, the evolution of glass transition temperature (Tg) and the melting temperature peak (Tm) as hydrolysis proceeded was reported.

From the values of temperature reported in Table 2, it was observed that both Tg and Tm decreased for all the samples over the time of hydrolysis: their values remained higher for 4032D + 3% (LDH-NO_3_).

The DSC results indicated that the presence of 3% (LDH-NO_3_) as filler in PLA made the samples more resistant to hydrolysis. On the contrary, the use of (LDH-CO_3_) as a filler accelerated the hydrolysis process of PLA. This material will therefore not be considered in the following.

#### 3.1.4. Gel Permeation Chromatography (GPC Analysis) 

GPC analysis were performed on 4032D and on 4032D + 3% (LDH-NO_3_). In order to evaluate any degradation phenomena of the extrusion process on the PLA, GPC analyses were performed on the non-hydrolyzed extruded PLA and PLA pellets. The average molecular weight of pure PLA after extrusion (Mn = 70 kDa; Mw = 102 kDa) was very similar to that of pellets (Mn = 75 KDa; Mw = 106 KDa), which demonstrated that the extrusion process induces a negligible degradation on pure PLA.

**Figure 5:**
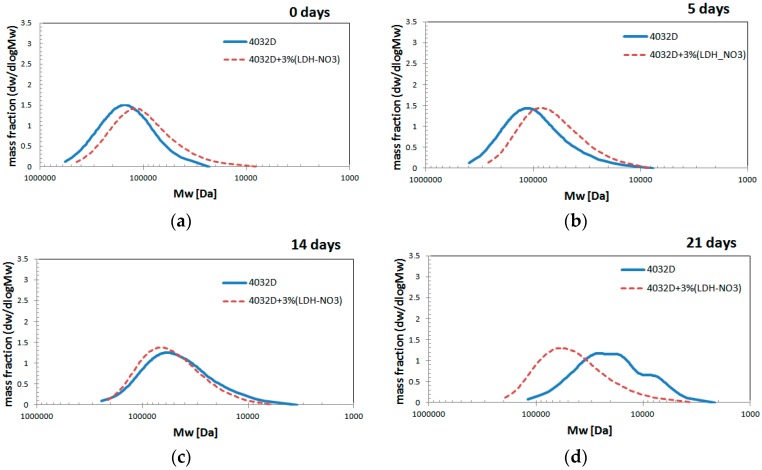
Evolution of molecular weight distribution during hydrolysis process (PLA and PLA + 3% LDH-NO_3_) at different hydrolysis times: (**a**) 0 days; (**b**) 5 days; (**c**) 14 days; (**d**) 21 days.

**Figure 6:**
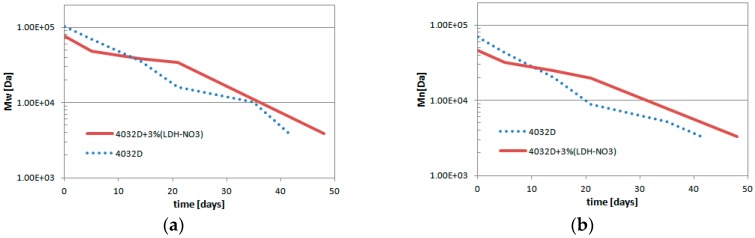
Variation of (**a**) weight average molecular (Mw) and (**b**) number average molecular weight (Mn), during the hydrolysis process (PLA and PLA + pure LDH).

The results of GPC analysis on hydrolyzed samples are reported in Figure 5 and Figure 6.

It can be observed that the curves related to both materials were shifted towards smaller molecular weights with ongoing hydrolysis. In particular, the molecular weight distribution curves related to PLA with LDH for the first five days were characterized by lower molecular weights than pure PLA ones: probably due to a degradation in the molten state during processing. From day 14 onwards, it was observed that the distribution of the molecular weight of the PLA with LDH was still being shifted toward smaller molecular weights, but more slowly than that of pure PLA. In fact, the molecular weight distribution curve of the PLA was located at lower values than that of PLA with LDH. The data confirm that LDH platelets protect PLA from the phenomenon of hydrolysis. After 21 days of hydrolysis (Figure 5d) a change of shape was observed for pure PLA, which presented a multimodal distribution. This particular phenomenon was probably due to the fact that the hydrolysis proceeded for all the polymer chains, but the presence of a significant crystalline content caused a slowing of the hydrolysis with respect to what occurred in the amorphous phase, thus leading to the formation of more peaks discriminated by their molecular weight distribution curve.

Such a positive effect was also evidenced in Figure 6, which reported on the variations of Mw (Da) and Mn (Da) of the two samples as function of the hydrolysis time (days). As a consequence of the hydrolytic process, the polymeric chains underwent a breakdown, with the molecular weight decreasing. The PDI index showed very small variation with hydrolysis time; in fact, the data shown in Figure 6a,b were very similar to each other. Mn and Mw values were higher for 4032D + 3% (LDH-NO_3_) with respect to pure PLA.

### 3.2. Experimental Results: LDH-Organic Acid Selection

Once the benchmark LDH was selected, namely LDH-NO_3,_ the effect of intercalation of organic acids was investigated.

Three different organic acids were taken in consideration: succinic acid, fumaric acid, and ascorbic acid; all of them were intercalated into LDH so that three different samples were analyzed:

4032D + 3% LDH-succinic acid (4032D + 3% LDH-succ)

4032D + 3% LDH-fumaric acid (4032D + 3% LDH-fum)

4032D + 3% LDH-ascorbic acid (4032D + 3% LDH-asc)

The experimental results of hydrolysis process in the solid state at 58 °C under the conditions previously mentioned are reported below. 

#### 3.2.1. Hydrolysis at High Temperature (Rheological Analysis)

**Figure 7:**
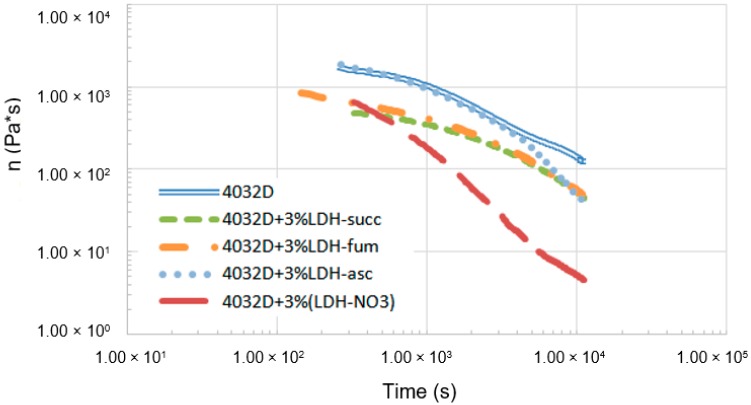
Time evolution of the complex viscosity during rheological tests of not hydrolyzed samples (PLA and PLA + 3% LDH-organic acids). The time on the horizontal axis includes the period needed to set the gap for each sample.

In Figure 7, the results of the rheological analysis are reported. The rheological tests were carried out in order to assess the significance of hydrolysis in the molten state. Time sweep test were performed on 4032D and on 4032D + 3% (LDH-organic acids).

From the data reported above, it can be observed that all materials presented a decrease of viscosity during time, due to hydrolysis and thermal degradation. Intercalating LDH with acids, allowed for the significant reduction of the decrease of viscosity that takes place when pure LDH is used as filler. This was a clear advantage for processing: it could be stated that the use of LDH with intercalated acids does not induce a dramatic degradation in the molten state, which can make the materials impossible to be processed.

#### 3.2.2. Weight Loss

**Figure 8:**
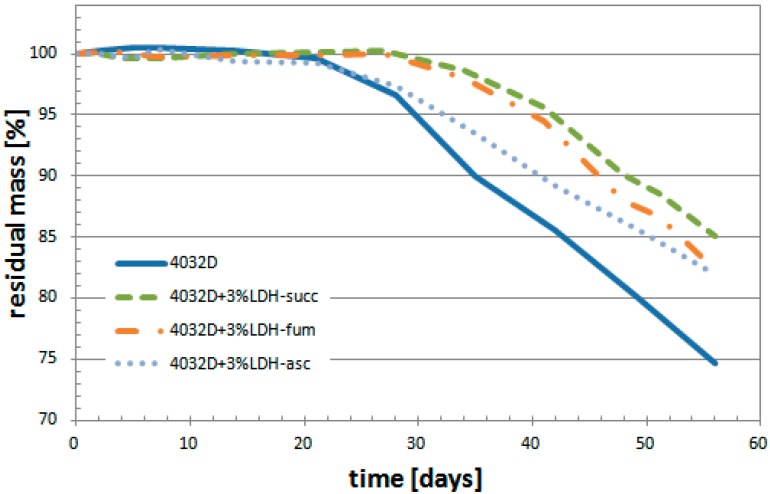
Weight loss during hydrolysis process (PLA and PLA + 3%LDH-organic acid).

In Figure 8, the variation of the sample mass is reported: for all the samples, a reduction of residual mass due to hydrolysis process was observed.

The presence of 3% of LDH-organic acid can preserve PLA from the hydrolysis process; in particular, this is even better exemplified with LDH-succ, which presents the best relative positive effect, as the time corresponding to its weight loss of 10%, t_10%_, is about 50 days compared to t_10%_ for a PLA of 35 days only. In summary, for the same residual mass, the time increased by about 40%.

#### 3.2.3. Calorimetric Analysis (DSC)

**Figure 9:**
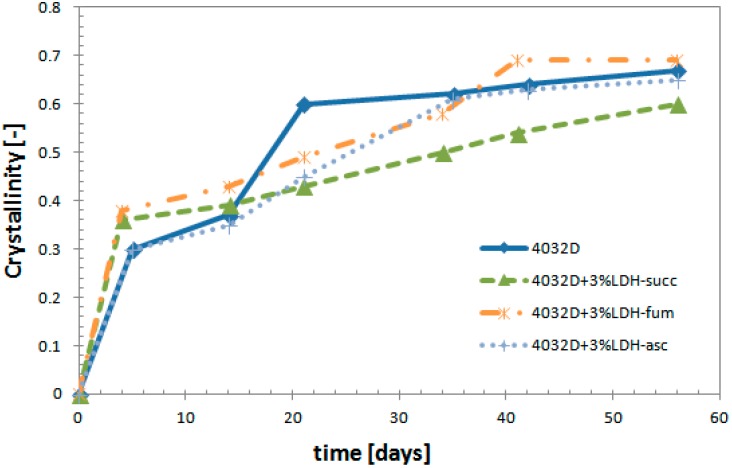
Variation of crystallinity degree during the hydrolysis process (PLA and PLA + 3% LDH-organic acid).

In Figure 9, the variation of crystallinity degree (Xc) as function of hydrolysis time is reported.

The different curves have a similar trend up to about 15 days, and then Xc of pure PLA increased rapidly vs time, while Xc of PLA + 3% LDH-organic filler increased more steadily vs. time, with a slope that was lower in the case of PLA + 3% LDH-succ.

As the hydrolysis proceeded, the polymer chains broke down and become smaller. Accordingly, the glass transition temperature (Tg) and the highest temperature peak of the melting during the first round of heating presented smaller and smaller values as a function of the time of hydrolysis. From the data reported in Table 3, it is clear that Tg and Tm decreased for all the samples. For the sample with 3% LDH-organic acid, this decrease was however, less pronounced than for pure PLA and for 4032D + 3% (LDH-NO_3_) (Table 2). What was observed happened in particular for PLA + 3% LDH-succ. DSC analysis results confirm what is observed from weight loss analysis.

#### 3.2.4. Gel Permeation Chromatography (GPC Analysis)

**Figure 10:**
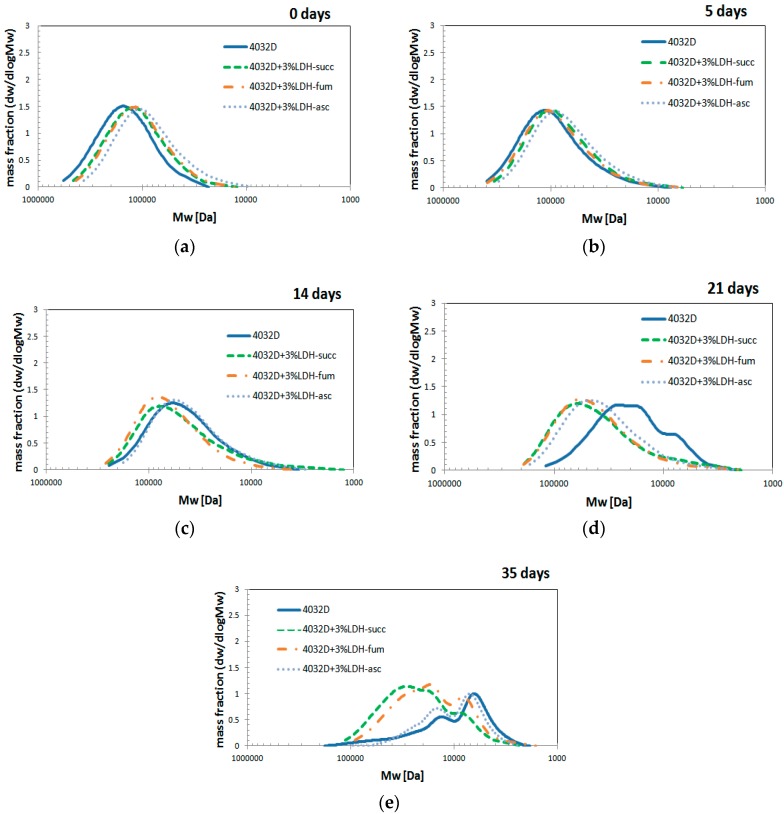
Evolution of molecular weight distribution during hydrolysis process (PLA and PLA+ 3% LDH-organic acid) at different hydrolysis time: (**a**) 0 days; (**b**) 5 days; (**c**) 14 day; (**d**) 21 days; (**e**) 35 days.

The molecular weight distribution curves of the sample analyzed by GPC at a determined hydrolysis time are displayed in Figure 10.

From Figure 10, it is possible to notice that the molecular weight distribution curves were shifted towards lower molecular weights as hydrolysis proceeded. As observed above, a change of shape was observed upon time, from monomodal to multimodal distribution.

No differences could be observed among the different samples at shorter times, but from 21 days onwards, the molecular weight distribution curves of samples with 3% LDH-organic acid remain at higher molecular weights with respect to pure PLA.

**Figure 11:**
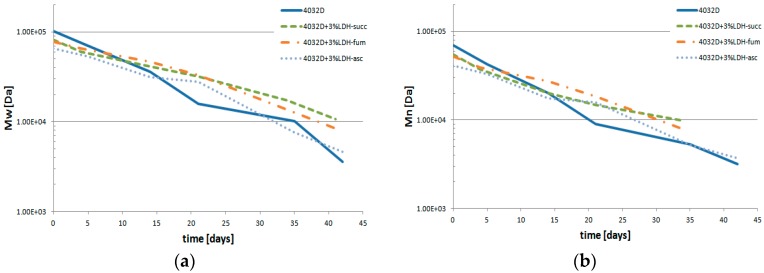
Variation of (**a**) the weight average molecular weight (Mw) and (**b**) the number average molecular weight (Mn) during the hydrolysis process of PLA and PLA + 3% LDH-organic acids).

In Figure 11a,b, the variations of weight average molecular weight (Mw) and of number average molecular weight (Mn) were reported. As observed above, for all samples, the molecular weights decreased, but all samples with 3% LDH-organic acids maintained a higher molecular weight with respect to the neat PLA and 4032D + 3% (LDH-NO_3_) (Figure 6) upon hydrolysis time. Furthermore, it can be noticed that the data of Mn and Mw reported in Figure 11 were very similar for both parameters, meaning that the PDI index remained quite unchanged.

#### 3.2.5. Analysis of PLA Hydrolysis Reaction: Reaction Rates

Briefly let us recall the mechanism of the hydrolysis reaction. The PLA hydrolysis reaction can be written as:

$$H_{2}O+\mathrm{ester}⇆-\mathrm{COOH}+-\mathrm{OH}$$

The kinetics of the hydrolysis process can be described by an autocatalytic mechanism [36]:

(2) $$\frac{{\mathrm{dC}}_{C}}{\mathrm{dt}}={\mathrm{kC}}_{C}C_{E}C_{H2O}$$

Were C_C_ is the carboxylic end-group concentration, and C_E_ is the ester concentration.

C_C_ and C_E_ can be related to the molecular weight as follows [37]: Equation (2):

(3) $$C_{C}=\frac{\rho }{M_{n}}$$

(4) $$C_{E}=\frac{\rho }{M_{n}}\left(\mathrm{DP}-1\right)$$

In these equations, ρ is the density of the polymer sample (about 1210 g/m^3^) and DP is the average degree of polymerization, defined as the ratio Mn/M (M is the molecular weight of the repeating unit, equal to 72 g/mol in our case). Equation (3) takes into account the number of ester linkages in the monomer unit in the case of the polyesters studied in this work.

After substituting Equations (3) and (4) into Equation (2) and rearranging, also considering the definition of DP, we obtain:

(5) $$\frac{\mathrm{dln}\left(M_{n}-M\right)}{\mathrm{dt}}=-k^{\prime }$$

(6) $$k^{\prime }=k\left[H_{2}O\right]\frac{\rho }{M}$$

k′ can be considered as the kinetic constant of the hydrolysis process and calculated from the time dependence of the number of the average molecular mass of the sample.

**Figure 12:**
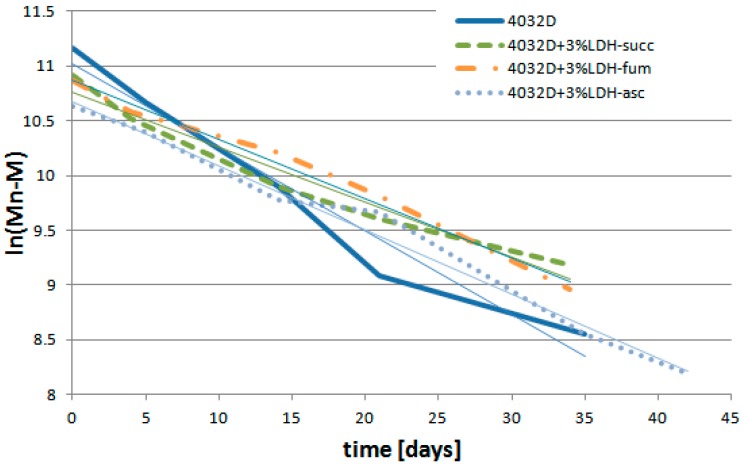
Variation of ln (Mn−M) during hydrolysis process (PLA and PLA + 3% LDH-organic acids).

In Figure 12, ln(Mn−M) is reported as a function of hydrolysis time:

The data of each sample in the figure above showed an approximately linear trend (the coefficients of reliability reported in Table 4 were quite close to 1). The slope of the lines constitutes the value of the kinetic constant, k′. A linear dependence versus time means that a unique value of k′ is extrapolated for each system. Since the kinetics constant of hydrolysis depends on the pH value of the medium, a single k′ value indicated that the pH is mostly constant in time. Correlatively, any irregularity in the slope is indicative of a slight change in k′ and so of the pH (as observed for PLA after 20 days). Remarkably, a quasi-constant k′ was observed for the PLA–LDH composites, most probably due to the controlled release of the organic acids intercalated in LDH. In Table 4, all the values of k′ and the coefficients of determination are reported.

The kinetics constant of PLA + 3% LDH-organic acid was lower than that of the neat PLA, and among PLA–LDH composites, 4032D + 3% LDH-succ exhibited the lowest value, as well as being the sample that was more resistant to hydrolysis process, thus confirming the GPC analysis (Figure 10).

#### 3.2.6. Mechanical Tests

Mechanical tests were performed on hydrolyzed samples: the penetration test, in order to evaluate if the samples more resistant to hydrolysis process were really more mechanically resistant.

**Figure 13:**
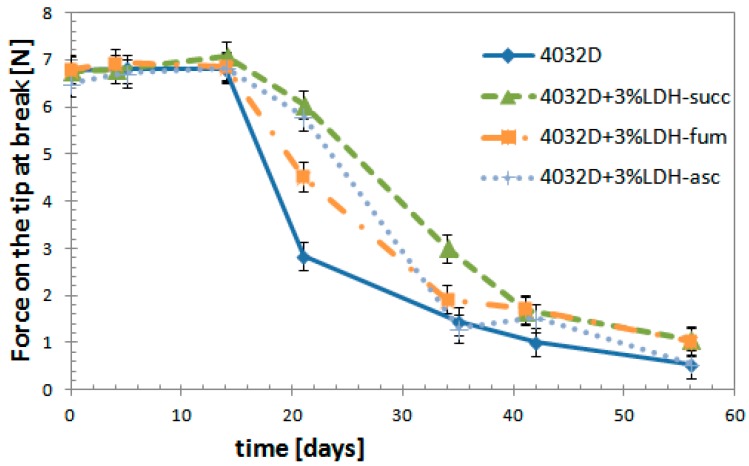
Variation of force at break during hydrolysis process (PLA and PLA + 3% LDH-organic acids).

From the Figure 13 it can be observed that up to about 15 days, where the force value remained constant at about 7 N, there were no fractures in all the samples. After 21 days, the samples began to break with decreasing amounts of mechanical stress as the hydrolysis proceeded. For the 4032D + 3% LDH-organic acid, the values of the force at break were higher than for neat PLA; in particular for 4032 + 3% LDH-succ. For this sample, the same reduction of force at break was found at times to be more than 30% longer with respect to pure PLA.

The mechanical analysis confirmed our previous analysis. Indeed, a dispersion of 3% LDH-organic acid delayed PLA strongly from the hydrolysis process, the sample that was more resistant to hydrolysis was with the composition filler LDH-succ.

#### 3.2.7. Visual Analysis of Hydrolyzed Samples

**Figure 14:**
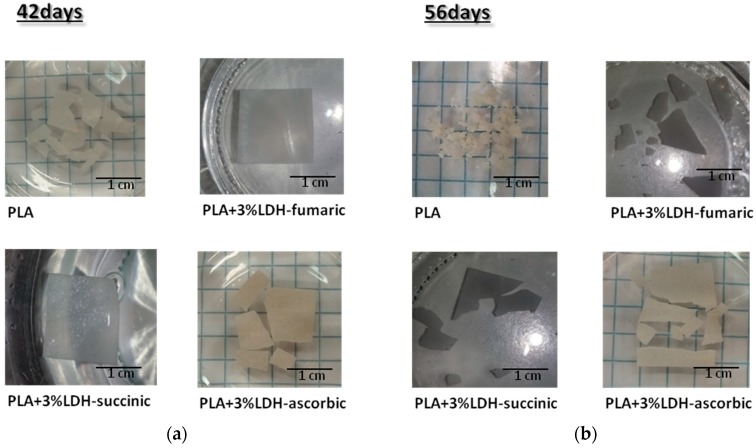
Images of hydrolyzed samples (PLA and PLA + 3% LDH-organic acids); (**a**) hydrolyzed at 42 days; (**b**) hydrolyzed at 56 days.

It is interesting to note that all previous analyses were somehow in agreement from a macroscopic point of view. In Figure 14, the photographs of the samples hydrolyzed at different times are displayed.

The protective effect of the addition of 3% LDH-organic acids to PLA against hydrolysis process was clearly visible. After 42 days, the sample remained intact for PLA composites with LDH-fum and -succ, this was even more pronounced after 56 days, time when PLA was entirely broken down.

## 4. Conclusions

The aim of this work was to obtain nano(bio)composites with a degradation rate that can be modulated in a limited amount of time. Eco-friendly and bio-compatible additives were adopted to modulate the degradation rate of PLA. The fillers were added to the polymeric matrix using an extrusion process, and subsequently, composite films were obtained by compression molding. The impact of hydrolysis was scrutinized by:

1. The weight variation of samples:

The presence in PLA of LDH-CO_3_ decreases the stability of the material with respect to hydrolysis, while it is found more resistant when using LDH-NO_3_ as filler. Adding 3% of LDH-organic acid can preserve PLA from the hydrolysis process, in particular LDH-succ presents the best protective effect.

2. Crystalline degree and glass transition temperature variations:

As the hydrolysis proceeds, an increase in the crystalline degree is observed. The crystalline degree can increase due to the occurrence of two phenomena—a decrease in the amount of the amorphous phase present in the sample (erosion by hydrolysis), and a crystallization of some chain segments from the amorphous phase. Xc of pure PLA increases quickly with respect to PLA + LDH-organic acids, while Xc of 4032 + 3% LDH-organic filler increases but still remains at lower values.

Regarding the glass transition temperature, it is noted that it decreases for all the samples with the progression of hydrolysis: their values remain higher for 4032D + 3% (LDH-NO_3_) with respect to pure PLA and 4032D + 3%(LDH-CO_3_) (Tg is higher by about 6 °C). The same differences are observed for PLA + 3% LDH-organic filler: all loaded samples show a higher Tg at the end of hydrolysis tests, in particular, 4032 + 3% LDH-succ.

3. Molecular weights variations

The average molecular weights of the analyzed samples decrease with the progress of the hydrolysis process. Mn and Mw values are higher for 4032D + 3% (LDH-NO_3_) with respect to pure PLA: therefore, the presence of LDH-NO_3_ as filler truly protects PLA from hydrolysis process. All samples with 3% LDH-organic acids as fillers keeps higher molecular weight with respect to neat PLA versus increased hydrolysis; in particular 4032D + 3% LDH-succ, which shows the lowest value of the kinetic constant of the hydrolysis process.

4. Mechanical tests

The strength at break shows a decrease in the ongoing of hydrolysis. After 21 days, the samples begin to break at decreasing mechanical stress as the hydrolysis proceeds. For the 4032D + 3% LDH-organic acid, the force at break is higher than that of neat PLA; in particular, for 4032 + 3% LDH-succ, thus confirming the previous analyses.

To summarize, all the characterizations concur well in demonstrating the efficiency of LDH filler in content as low as 3 wt % to delay the hydrolytic degradation of PLA. Among the studied samples, LDH-succ filler appears as a promising candidate to significantly delay the breakdown of PLA. It is our belief that such a combination between food additive and benign inorganic vessel may pave a new route in designing PLA composites for targeted applications, which can range from durable ones, such as automotive parts or equipment housing, to biological contacts such as implants that need a given degradation rate, or agricultural materials.

## Figures and Tables

**Table 1:** Extruded materials.

Groups	Extruded Materials (Filler 3 *w*/*w*)
Pure PLA	4032D
PLA + LDH	4032D + (LDH-CO_3_)
	4032D + (LDH-NO_3_)
PLA + (LDH-organic acids)	4032D + LDH-succinic acid
	4032D + LDH-fumaric acid
	4032D + LDH-ascorbic acid

**Table 2:** Summary of DSC results: Tg was measured during the second heating scan, the reported Tm refers to the highest-temperature peak of the melting endotherm during the first heating ramp (PLA and PLA + pure LDH).

Sample		Days of Hydrolysis				
		0	5	14	21	48
4032D	Tg (°C)	63.8	63.7	61.4	61.4	51
	Tm (°C)	169.9	168.4	167.8	164.9	154.3
4032D + 3% (LDH-CO_3_)	Tg	61.8	/	/	/	50.2
	Tm	166.4	164.8	162.9	158.6	150.3
4032D + 3% (LDH-NO_3_)	Tg	63.3	62.9	62.2	62.5	56.9
	Tm	168.2	167.5	166.8	166	155.3

**Table 3:** Summary of DSC results: Tg was measured during the second heating scan, and the reported Tm refers to the highest temperature peak of the melting endotherm during the first heating ramp (PLA and PLA + 3% LDH-organic acid).

Sample		Days of Hydrolysis				
		0	5	14	21	48
4032D	Tg (°C)	63.8	63.7	61.4	61.4	51
	Tm (°C)	169.9	168.4	167.8	164.9	154.3
4032D + 3% LDH-succ	Tg	62.8	62.3	61.8	61.7	56.6
	Tm	169.8	167	166.7	166.5	162.5
4032D + 3% LDH-fum	Tg	62.2	61.5	61.1	60.9	56.1
	Tm	169.2	166.9	166.7	166.5	161
4032D + 3% LDH-asc	Tg	61.2	61.1	59.1	58.6	55.8
	Tm	169.6	168	167.4	167.3	158.2

**Table 4:** Kinetic constant of hydrolysis process (PLA and PLA + 3% LDH-organic acids).

	k′ (Days^−1^)	R^2^
4032D	0.0765	0.9628
4032D + 3%LDH-succ	0.0503	0.9591
4032D + 3%LDH-fum	0.0543	0.9871
4032D + 3%LDH-asc	0.0587	0.9868

## Supplementary Materials

The following are available online at http://www.mdpi.com/1996-1944/11/10/1943/s1, Figure S1: Powder XRD patterns of acids-LDH filler materials.
