# Supplementary material for: Poly(Lactic Acid)-Based Nanobiocomposites with Modulated Degradation Rates
Source: Materials (Basel). 2018 Oct 11;11(10):1943. doi: 10.3390/ma11101943 (PMC6213022; doi:10.3390/ma11101943)
Supplement: Supplementary file 1 [file materials-11-01943-s001.pdf]

materials

Supplementary

Poly (Lactic Acid)-Based Nanobiocomposites with Modulated Degradation Rates

Iozzino Valentina [1], Askanian Haroutioun [2], Leroux Fabrice [2], Verney Vincent [2,*] and Pantani Roberto [1,*]

[1] Department of Industrial Engineering, University of Salerno Via Giovanni Paolo II, 132, 84084 Fisciano (SA), Italy; viozzino@unisa.it

[2] Institut de Chimie de Clermont Ferrand (ICCF), UMR 6296 Université Clermont Auvergne, CNRS, Sigma Clermont, ICCF, F-63000 Clermont-Ferrand, France; haroutioun.askanian@sigma-clermont.fr (A.H.); Fabrice.Leroux@uca.fr (L.F.)

* Correspondence: Vincent.Verney@uca.fr (V.V.); rpantani@unisa.it (P.R.)

Received: 30 August 2018; Accepted: 5 October 2018; Published: 11 October 2018

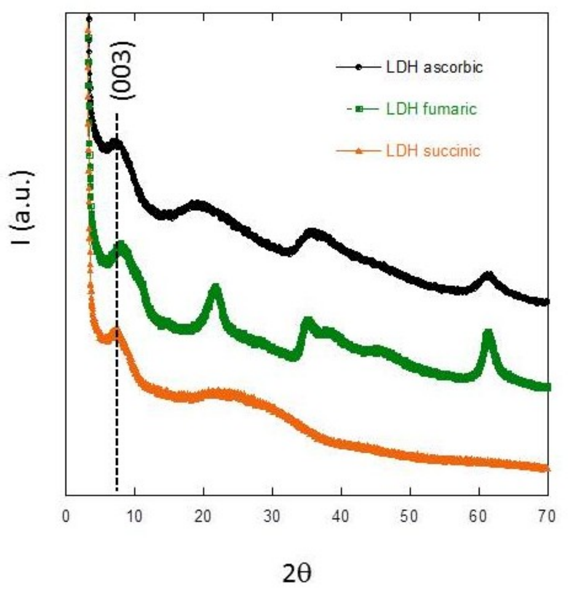

Figure S1. Powder XRD patterns of acids-LDH filler materials.

© 2018 by the authors. Submitted for possible open access publication under the terms and conditions of the Creative Commons Attribution (CC BY) license (http://creativecommons.org/licenses/by/4.0/).
